# Primary small rectal neuroendocrine tumor with pelvic lateral lymph nodes metastasis: A case report and review of literature

**DOI:** 10.1016/j.ijscr.2025.110963

**Published:** 2025-01-30

**Authors:** Lihong Li, Ziyue Chen, Dajian Zhu, Qianbao Lv, Tianpeng Zhang, Jinsong Lai

**Affiliations:** a Department of Gastrointestinal surgery, Shunde Women and Children's Hospital of Guangdong Medical University (Maternal & Child Healthcare Hospital of Shunde Foshan), Foshan 528300, China; b Postgraduate of Guangdong Medical University, Zhanjiang 524002, China; c Department of Gynaecology, Shunde Women and Children's Hospital of Guangdong Medical University (Maternal & Child Healthcare Hospital of Shunde Foshan), Foshan 528300, China

**Keywords:** Neuroendocrine tumor, Rectum, Lateral lymph node dissection, Local resection, Total mesorectal excision, Case report

## Abstract

**Introduction and importance:**

Neuroendocrine tumors (NETs) can occur in almost any organ of the body, and they vary in size and volume. The small tumors may be <1 cm in size, but the large ones can exceed 20 cm. The probability of developing NETs in different organs varies greatly, but rectal NETs are relatively common. Our aim is to tell the surgeon that rectal NETs metastasize to the pelvic lateral lymph nodes even in the early stages and small sizes.

**Case presentation:**

In this report, we present the case of a 47-year-old man who was asymptomatic and incidentally diagnosed with a small NET in the rectum during an enteroscopy for physical examination. The diagnosis was confirmed by pathological examination of the biopsy. The CT of the patient was performed pre-hospital for daily physical examination, but nothing could be seen in the rectum from the CT. Then, the diagnostic local resection and a wide free margin was performed on the patient, and a second operation (radical resection of rectal cancer and extensive lateral pelvic lymphadenectomy) was performed due to the post-operative pathological report and the discovery of enlarged pelvic lymph node by PET-CT (positron emission tomography CT). After the surgery, the patient is in good condition and have no other symptoms except for a slight feeling of defecating.

**Clinical discussion:**

NETs could metastasize to the pelvic lateral lymph nodes, if so, the Total mesorectal excision (TME) should be performed, and the invasion and metastatic spread in NETs have to do with the regulatory factor named snail1 and Foxc2. We find that there is no standardized treatment approach for rectal NETs, which should be neither overtreated nor undertreated as far as possible, so the timing of surgery plays an important role, and long-term follow-up of the patients is extremely important.

**Conclusion:**

The purpose of the presentation of this case is to highlight the potential for rectal NETs to metastasize to the pelvic lateral lymph nodes even in the early stages and small sizes, without muscular layer or neurovascular invasion, and lateral lymph node dissection were necessary, emphasizing the importance of timing for surgery.

## Highlights

• This case highlights the potential for rectal NETs to metastasize to the pelvic lateral lymph nodes even in the early stages, or when the depth of invasion is limited to the submucosal layer.
• There is no standardized treatment approach for rectal NETs,00 the timing of surgery plays a critical role, and long-term follow-up of the patients is very important.
• The high expression of snail1 and Foxc2 was associated with invasion and metastatic spread in neuroendocrine tumors.
• The lymph nodes of metastasis indicated a more severe progression than the primary tumor.

## 1 Introduction and importance

Neuroendocrine tumors (NETs) were previously considered rare lesions, but their incidence has been increasing steadily over the past decades due to advancements in endoscopy, pathological technology, endoscopic ultrasound, and other diagnostic tools. Many of these tumors are often discovered incidentally during physical examinations. NETs exhibit diverse biological behaviors, ranging from indolent growth to low-grade malignancy and high invasion and metastasis. In this report, we aim to present a case of a NET that exhibited lymph nodes metastasis without invasion of the muscular layer, despite its small size. We believe that this report holds great significance for the diagnosis and treatment of patients with NETs. This case report has been reported in line with the Surgical Case Report (SCARE) Criteria [1].

## 2 Case presentation

**Fig. 1:**
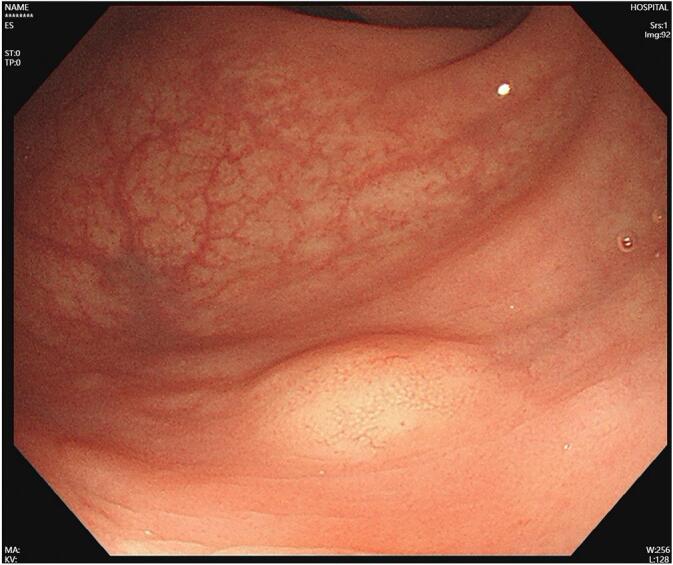
The tumor was 6 mm × 8 mm in the lower rectum about 5 cm from the anal verge.

**Fig. 2:**
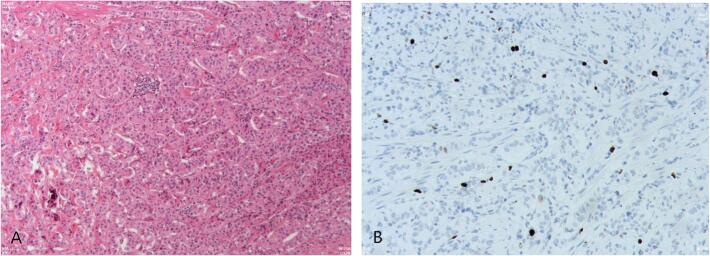
Histopathological and Immunohistochemical staining of the resected specimen (primary tumor) indicated that the tumor was neuroendocrine tumor, tumor tissue invaded the muscularis mucosae and fibrovascular tissue of submucosa, the Ki-67 index was 6 %. (Fig. A: Histopathological staining; Fig. B: Immunohistochemical staining).

**Fig. 3:**
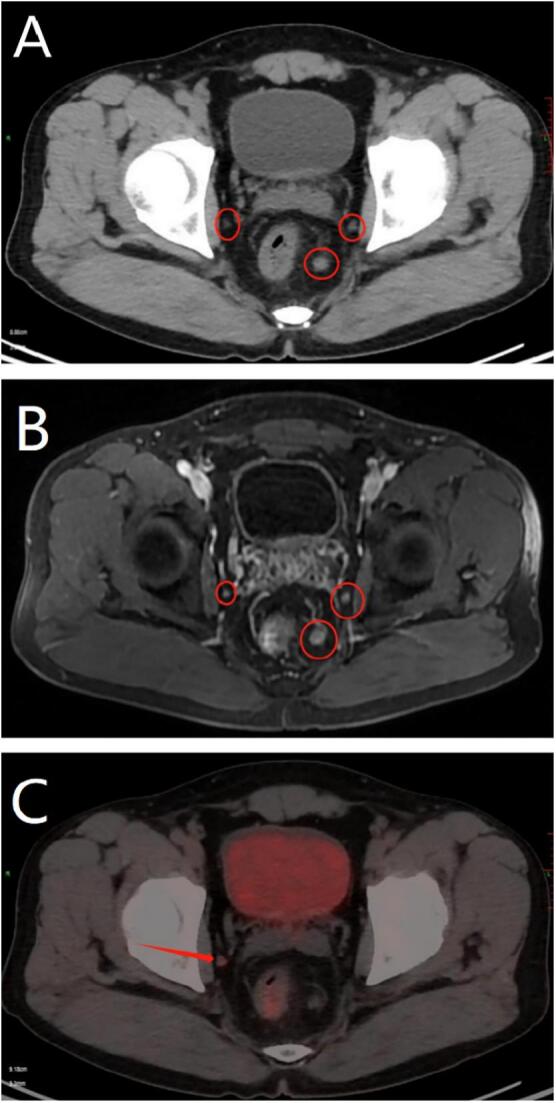
CT(Fig. A) and MRI(Fig. B) indicated many enlarged lymph nodes in the mesorectum and on the both sides of the lateral pelvic space(red circle). PET-CT(Fig. C) showed that the lymph nodes in the mesorectum was positive. (For interpretation of the references to colour in this figure legend, the reader is referred to the web version of this article.)

**Fig. 4:**
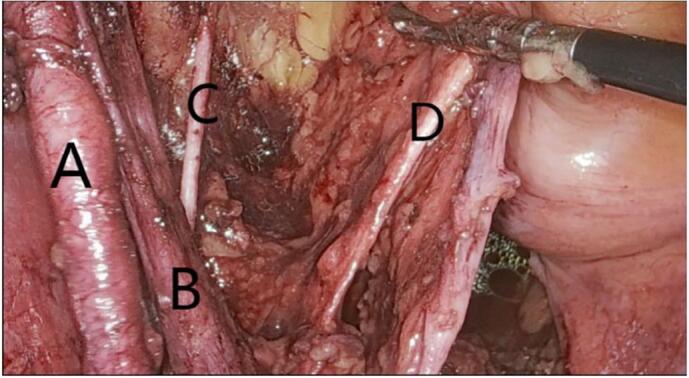
TME and lateral lymph node dissection were performed under laparoscopy. The figure showed that both sides of the lateral lymph nodes were removed. (A: the common iliac artery; B: the common iliac vein. C: the left obturator nerve; D: the right obturator nerve).

During a routine physical examination, a small rectal tumor measuring approximately 6 mm × 8 mm (Fig. 1) was accidentally discovered by colonoscopy in a 47-year-old man who was previously in good health, who had no any other diseases and no operation history before. The tumor was located about 5 cm from the anal verge on the left posterior side of the rectal wall. The biopsy confirmed the presence of a NET based on positive staining for CD56, CgA, Syn, and CK. The CT of the patient was performed pre-hospital for daily physical examination, but nothing could be seen in the rectum from CT. The patient was subsequently admitted to our hospital, then diagnostic local resection was performed through the anus, which included the entire submucosa and a small portion of the intrinsic muscular layer of the rectal wall. The pathological diagnosis revealed a NET G2, with a Ki-67 index of 6 % (Fig. 2) and a nuclear division count of 2/10HPF (high power field). The tumor was confined to the submucosal layer, and the margins were clear. Based on these findings, additional surgical resection was not deemed necessary, and the patient was discharged. However, approximately one month later, abdominal contrast-enhanced CT and T2-weighted MRI revealed enlarged lymph nodes (Fig. 3), raising suspicion of mesorectal lymph nodes and lateral lymph nodes metastasis on the left side. Somatostatin scintigraphy (DOTATATE imaging agent) identified 8 suspected metastatic lymph nodes, with the largest measuring 6 mm × 7 mm, located in the left side of the pelvic cavity. Thus the patient was readmitted to the hospital. Prior to the second operation, we conducted a case discussion and consider that the patient had NET with lymph nodes in the mesorectum and lateral lymph nodes metastasis and recommended treating with TME and bilateral lymph node dissection (Fig. 4).

Laparoscopic TME and bilateral lymph node dissection was performed as follows. Firstly, the original scar on the rectum, located approximately 5 cm from the anal verge, was marked using a small clamp under colonoscopy guidance. Ureteral stents were inserted on both sides to protect the ureters from injury. Lymph nodes around the inferior mesenteric artery (IMA), sigmoid artery, and superior rectal artery were dissected, with preservation of the IMA. The rectum was then cut and removed 1 cm above the dentate line, including the total mesorectum. The proximal cut of the colon was made 12 cm above the original scar. Subsequently, lateral lymphadenectomy was performed on both sides of the pelvic wall. Lymphatic and adipose tissue were dissected from the pelvic wall, including the internal iliac vessels, obturator vessels, pelvic plexus, and obturator nerves, all of which were preserved. The operation lasted for 330 min with a blood loss volume of approximately 200 mL. No blood transfusion was required during the surgery. Five days post-surgery, the patient experienced a slight increase in the frequency of defecation, approximately 4 times per day, without diarrhea or fecal incontinence. On the 10th day after surgery, the patient was discharged and no adjuvant chemotherapy was administered.

The pathological report indicated no residual tumor in the rectal mucosa specimen. Among the lymph nodes examined, 5 lymph nodes in the mesorectum contained metastases from the NET. Of the 22 left lateral lymph nodes, two were positive for metastases, while all 13 lateral lymph nodes on the right side were negative. Additionally, one lymph node adjacent to the left seminal vesicle showed positive.

The patient has been undergoing regular follow-up for several months since discharge, and his current condition is good. He does not exhibit any symptoms except for a slight feeling of defecation, with a frequency of twice a day. His sexual function is normal.

## 3 Clinical discussion

NETs are highly heterogeneous tumors that originate from neuroendocrine cells and can develop in various organs throughout the body. In recent years, the incidence of NETs has been on the rise, which is likely attributed to advancements in diagnostic technologies such as endoscopy, CT, MRI, and PET-CT. Rectal NETs were considered relatively rare in past [2]. However, in recent years, there has been an increase in the incidence of rectal NETs. This may be attributed to the widespread use of endoscopy for gastrointestinal carcinoma screening.

According to the National Comprehensive Cancer Network guidelines [3], rectal NETs measuring ≤2 cm in diameter can be treated with transanal or endoscopic resection and follow-up was necessary if the tumor was between 1 and 2 cm in size. A study [4]involving 86 patients with 90 rectal NETs (ranging from 2 mm to 13 mm in diameter) reported that all tumors were confined to the submucosal layer and classified as NET G1. No additional surgery was performed, and despite the presence of lymphatic and vascular invasion, there were no instances of metastasis or recurrence during a median follow-up period of 67.5 months. Thus for early-stage NETs, endoscopic resection is valid and the long-term prognosis is excellent. However, if the tumor is larger than 2 cm and invades the muscularis propria or is suspected of lymph nodes metastasis, treatment with low anterior resection or abdominoperineal resection is recommended [3].

**Fig. 5:**
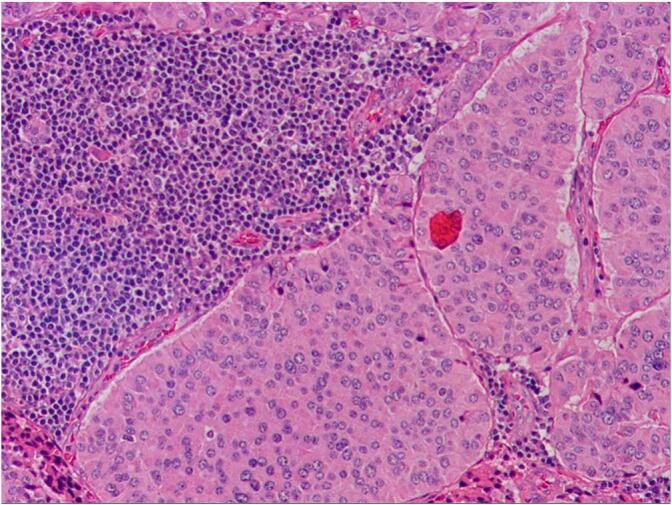
Hematoxylin and eosin staining for lateral lymph node showed that tumor was positive after the surgery of TME and lateral lymph node dissection (magnification, ×400).

**Fig. 6:**
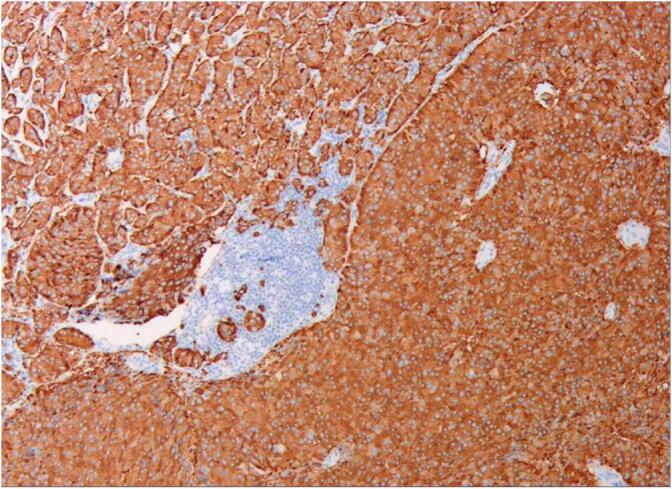
Immunohistochemical staining for lateral lymph nodes showed that Ki-67 index was 40 % after surgery of TME and lateral lymph node dissection, Synaptophysin(Syn,+), Chromogranin A(CgA,+), CD56(+), Cytokeratin20(CK20,-), (magnification, ×400).

In this case, the tumor measured 6 mm × 8 mm and limited to the submucosal layer without invasion into the muscularis propria according to the pathological report. There were no observed neural or vascular invasions, thus trans-anal local resection of the NETs was performed. One month later, additional surgery was necessary due to the detection of suspicious lymph nodes metastases in the mesorectum and both pelvic walls through somatostatin scintigraphy. Laparoscopic TME and bilateral lateral lymph node dissection were performed (Fig. 4). A total of 5 lymph nodes resected from the mesorectum were found to contain metastatic NETs. Among the 22 lateral lymph nodes on the left side, 2 were found to be metastatic, while all 13 lateral lymph nodes on the right side were negative. Pathological analysis confirmed a diagnosis of NET G3 for the lymph nodes metastasis in the mesorectum of the left side, based on a Ki-67 index of 40 % (Fig. 5, Fig. 6), indicating a more severe progression than the primary tumor in the rectum. However, no tumor remained in the inherent muscular layer of the rectum, suggesting that lymph node metastasis from the NETs could occur in the early stages. Fujii *et al* reported that metastasis to the lateral lymph nodes of the pelvic cavity may be associated with differences in the tumor's origin, and patients should undergo extended imaging examinations over an extended period of time. If suspicious lymph nodes are observed, radical resection of rectal cancer should be considered [5]. Our case is consistent with the findings of Fujii.

The mechanism of metastasis in NETs remains unclear and differs from that of common gastrointestinal tumors. Galvan *et al* reported that high expression of snail1 and Foxc2 was associated with invasion and metastatic spread in gastroenteropancreatic neuroendocrine tumors (GEP NETs) [6].Snail1 is known to be a regulatory factor of epithelial-mesenchymal transition (EMT), a biological process that enhances tumor cell migration. Additionally, a study including 750 patients with rectal NETs, indicated that rectal NETs with lymphovascular invasion had a significantly higher rate of regional lymph node metastasis, even in cases where the tumor size was <1.5 cm [7]. Some reports suggest that lymphovascular invasion may be present in a high percentage of small rectal NETs, but it may not have a significant impact on patient prognosis following resection [8]. We identified 9 reports covering 11 cases over the past decade (Table 1) [5,[9], [10], [11], [12], [13], [14], [15], [16]]. The tumor grade distribution was as follows: 6 cases were G1, 4 cases were G2, and the grade was unknown in one case. All cases showed lateral lymph node metastasis from NETs, with 5 cases (4 males and 1 female) indicating metastatic lymph nodes in the mesorectum. Various surgical procedures were performed in these patients (Table 1). In 5 cases, metastatic lateral lymph nodes were discovered and lateral lymph node dissection was performed at a later time after the initial surgery (one case had a 23-year interval). All patients were alive during the follow-up period (12–288 months).

**Table 1:** the reported cases of rectal NETs with LLN metastasis.

First author, year	Age/sex	Tumor size (mm)/Grade	Depth of invasion	Lymphovascular invasion	MLNM	Number/max size (LLNM), mm	Therapeutic measures	Recurrence time and interval	Prognosis	Refs
FUJII *et al,* 2020	47/M	10/G1	SM	+	−	1/7	ESD	36 m/TME + LLND	12 m/alive	[4]
Yoshitaro *et al*, 2013	65/M	10/Unknown	SM	+	+	1/13	LAR+TME	60 m/LLND	10 m/alive	[8]
Miyake *et al,* 2014	44/M	12/G2	SM	+	+	3/55	ISR + LLND	No	19 m/alive	[9]
Nakamoto, *et al,* 2014	70/M	20/G1	SM	−	+	1/unknown	LAR	50 m/LLND	72 m/alive	[10]
Beppu *et al,* 2016	59/M	7/G1	SM	+	−	1/7	ISR + TME + LLND	NO	36 m/alive	[11]
Umeda *et al,* 2016	66/M	7/G1	SM	−	−	1/23	TLR	276 m /LLND	288 m/alive	[12]
Tokumaru, *et al,* 2020	55/M	14/G2	SM	−	−	1/14	ISR + LND	54 m/LLND	96 m/alive	[13]
Shin *et al,* 2022	36/M	9/G1	SM	−	+	4/22	ISR + IMAL+LLND	NO	32 m/alive	[14]
Liu, *et al,* 2022	43/F	20/G2	SM	Unknown	+	1/9	ISR + LLND	12 m/liver metastases	24 m/alive	[15]
Liu *et al,* 2022	42/M	13/G1	−	Unknown	−	2/8	TLR + LLND	NO	22 m/alive	[15]
Liu *et al,* 2022	22/M	6/G2	SM	Unknown	−	1/10	TLR + LLND	NO	12 m/alive	[15]

M: male; F, femal; MLNM: mesorectal lymph node metastasis; LLNM: Lateral lymph node metastasis. m: month; LLND: lateral lymph node dissection; SM:submucosa;

LAR: low anterior resection. TME: total mesorectal excision; ISR: intersphincteric resection; TLR: transanal local resection; ESD: endoscopic submucosa dissection;

IMAL: inferior mesenteric artery ligation; LND: lymph node dissection;

## 4 Conclusion

In conclusion, this case highlights that rectal NETs can exhibit lateral lymph node metastasis even in the early stages, without muscular layer or neurovascular invasion. Therefore, performing laparoscopic TME and bilateral lymph node dissection is essential. It is important to note that there is no standardized treatment approach for rectal NETs. To avoid overtreatment or undertreatment, the timing of surgery plays a critical role, and long-term follow-up of the patients is extremely important.

## CRediT authorship contribution statement

LL and LQ and ChZ collected the patient clinical data, LL was responsible for the conception and design and wrote the manuscript. ZhD and LL performed the first surgery–tumor local dissection. ZhD, HH, ZhT, LJ performed the second surgery–TME and lateral lymph node dissection. ZhD revised the manuscript.

## Ethical approval

This study protocol was reviewed and approved by the Ethics Committee of our institution.

## Guarantor

Lihong Li and Dajian Zhu.

## Funding

This research did not receive any specific grant from funding agencies in the public, commercial, or not-for-profit sectors.

## Statement

The work has been reported in line with the SCARE criteria.

## Patient consent for publication

Written informed consent was obtained from the patient for publication and any accompanying images. A copy of the written consent is available for review by the Editor-in-Chief of this journal on request.

## Declaration of competing interest

The authors state that they have no conflicts of interest for this report.

## Data Availability

All data generated and analyzed during the course of this study are included in the article. Any further query could be addressed to the corresponding author.
